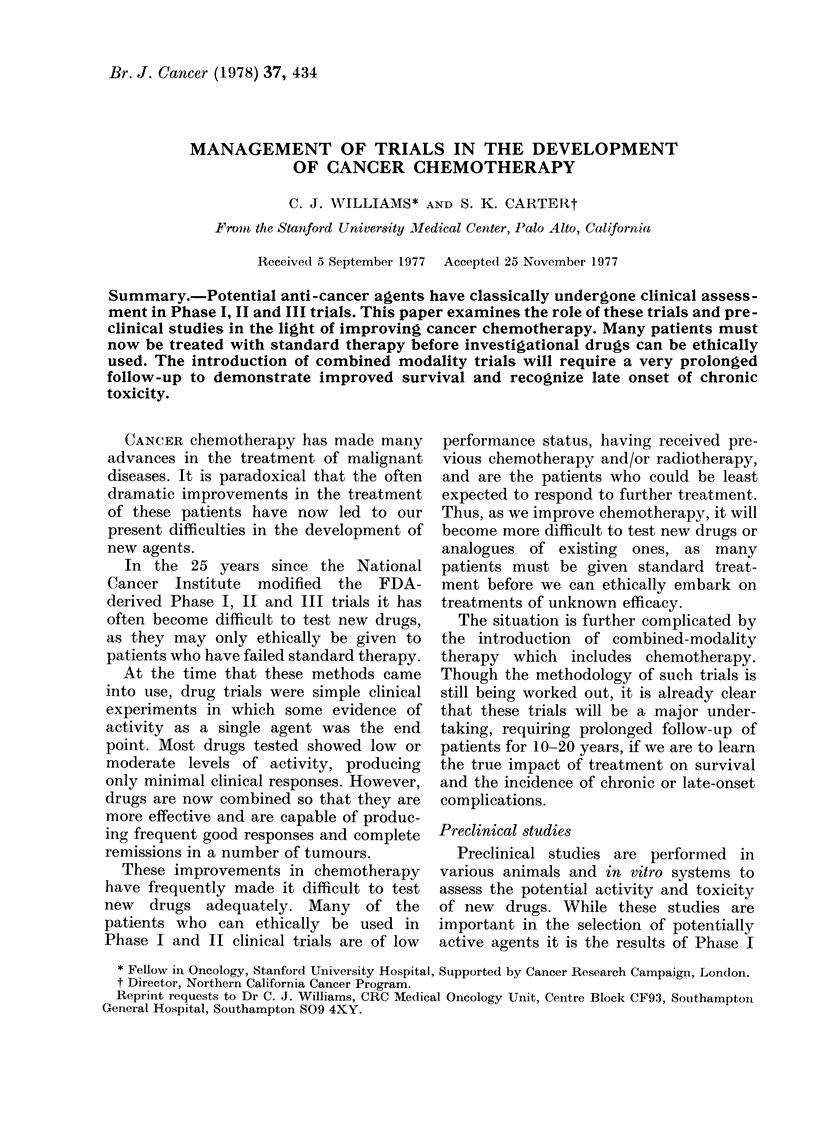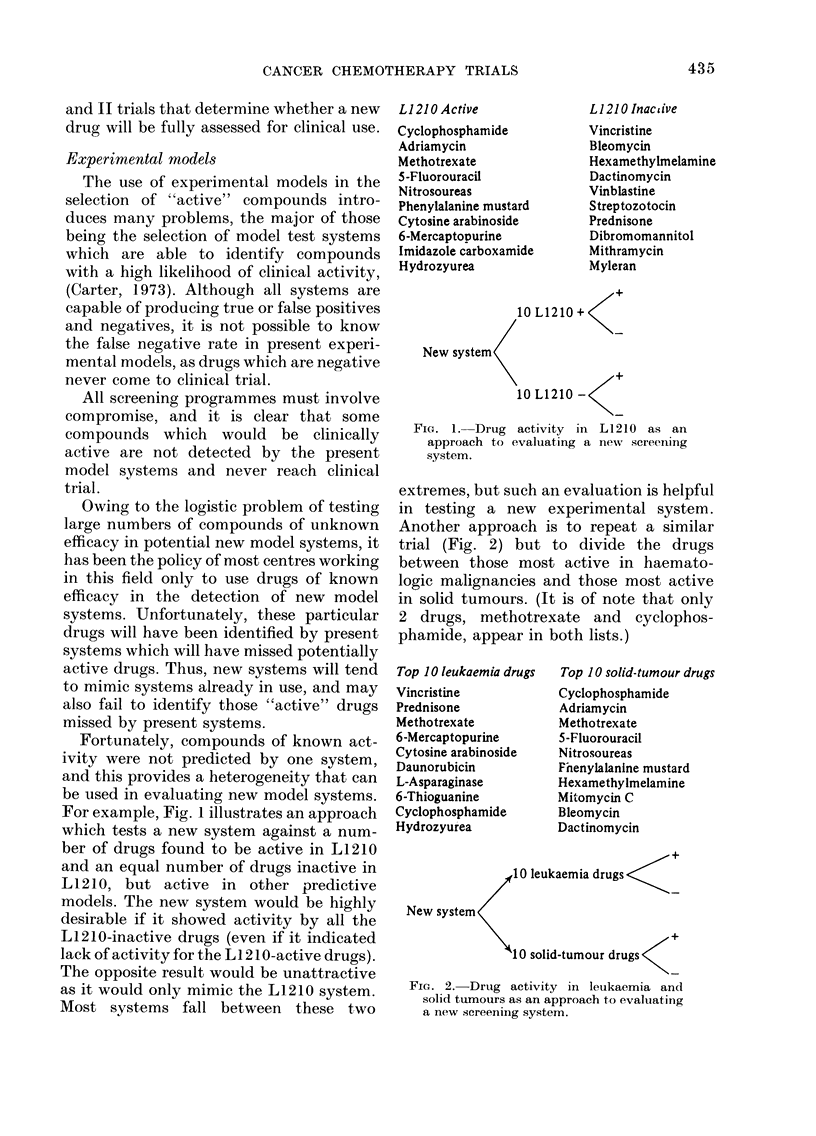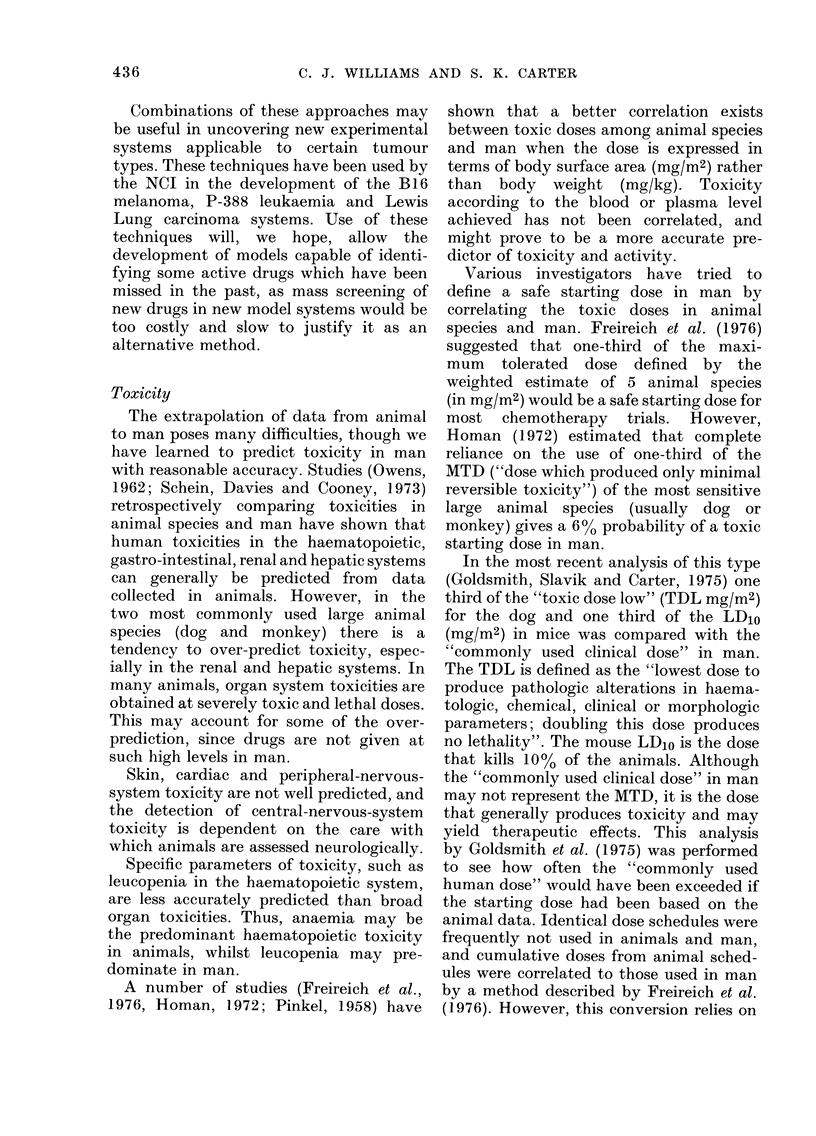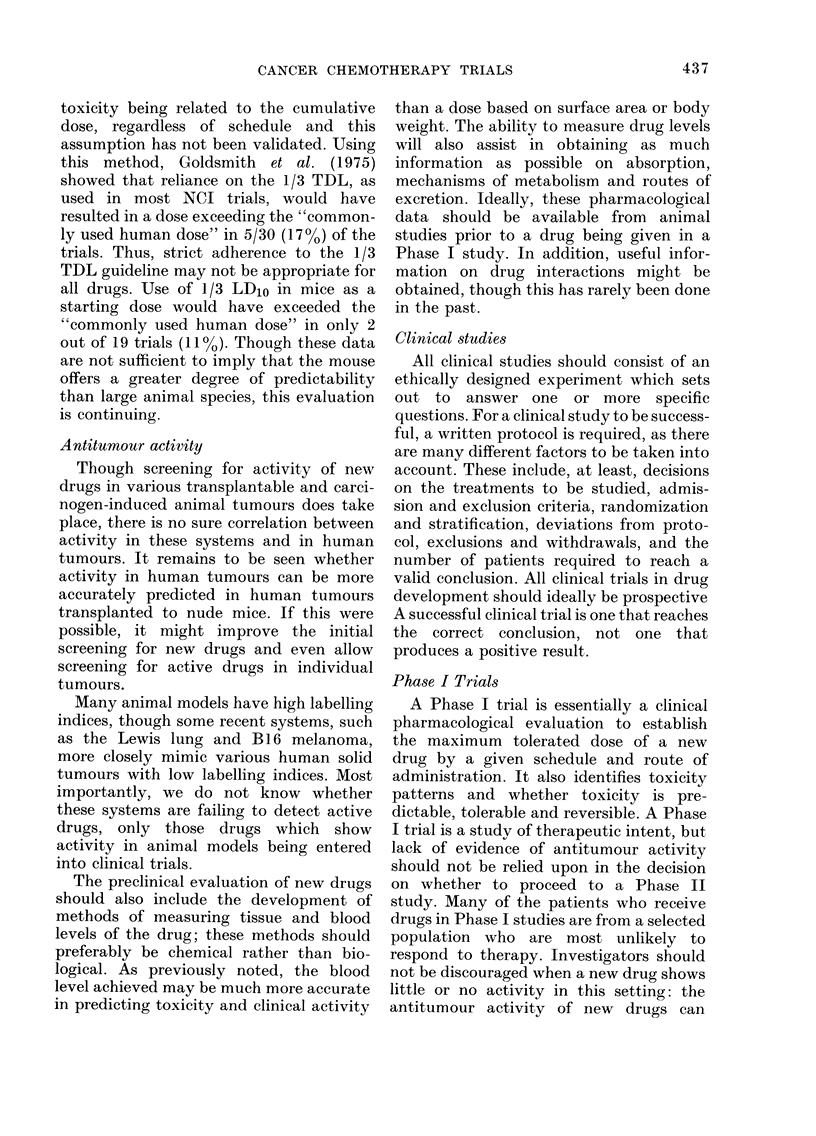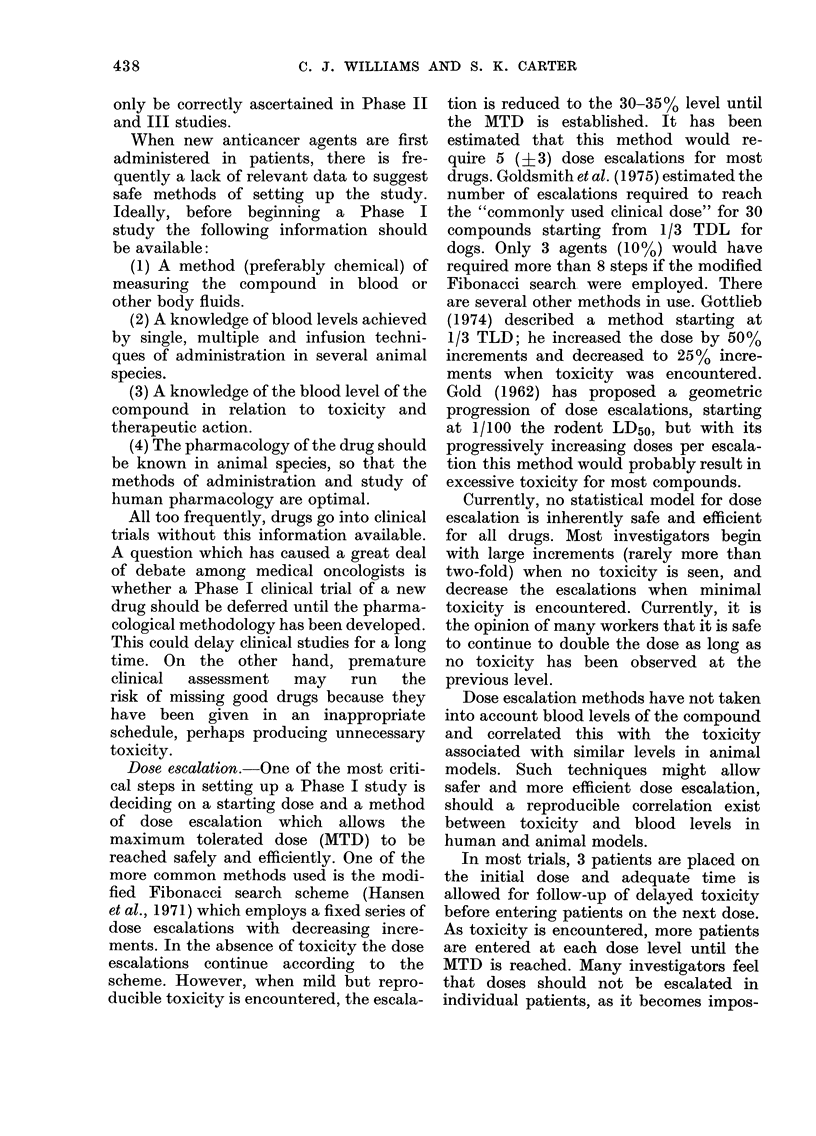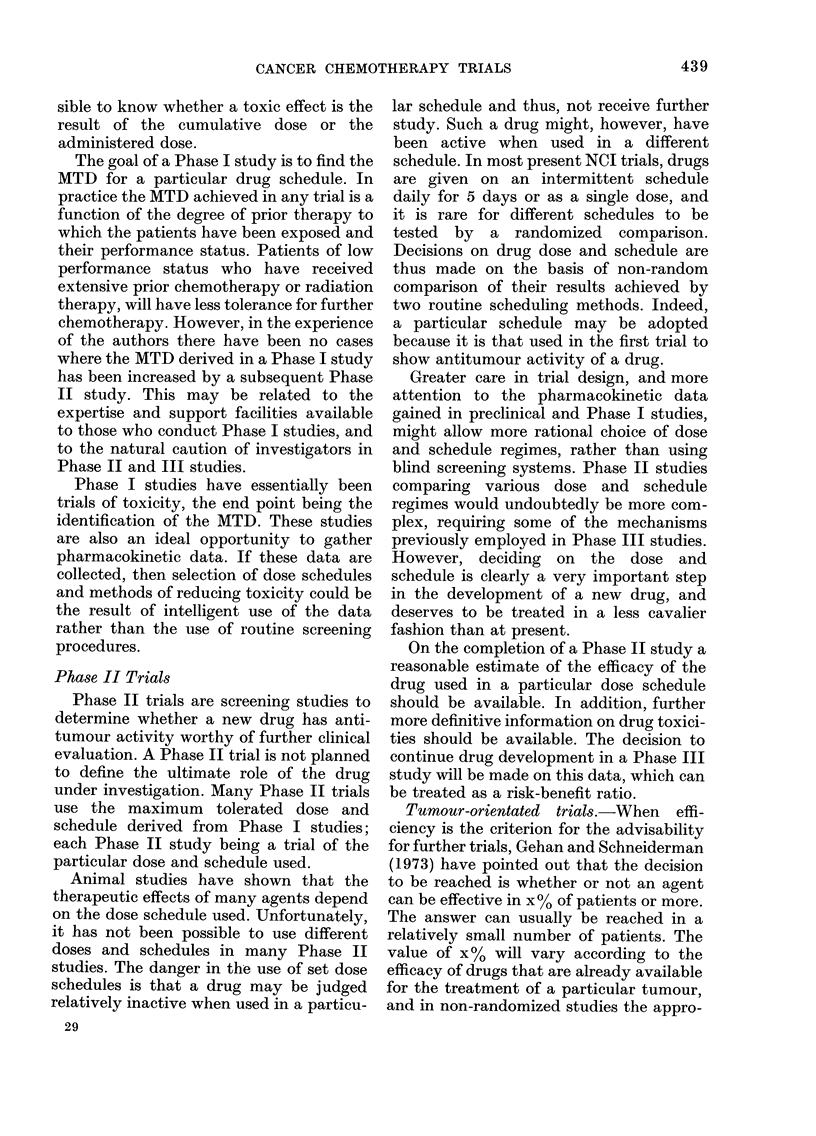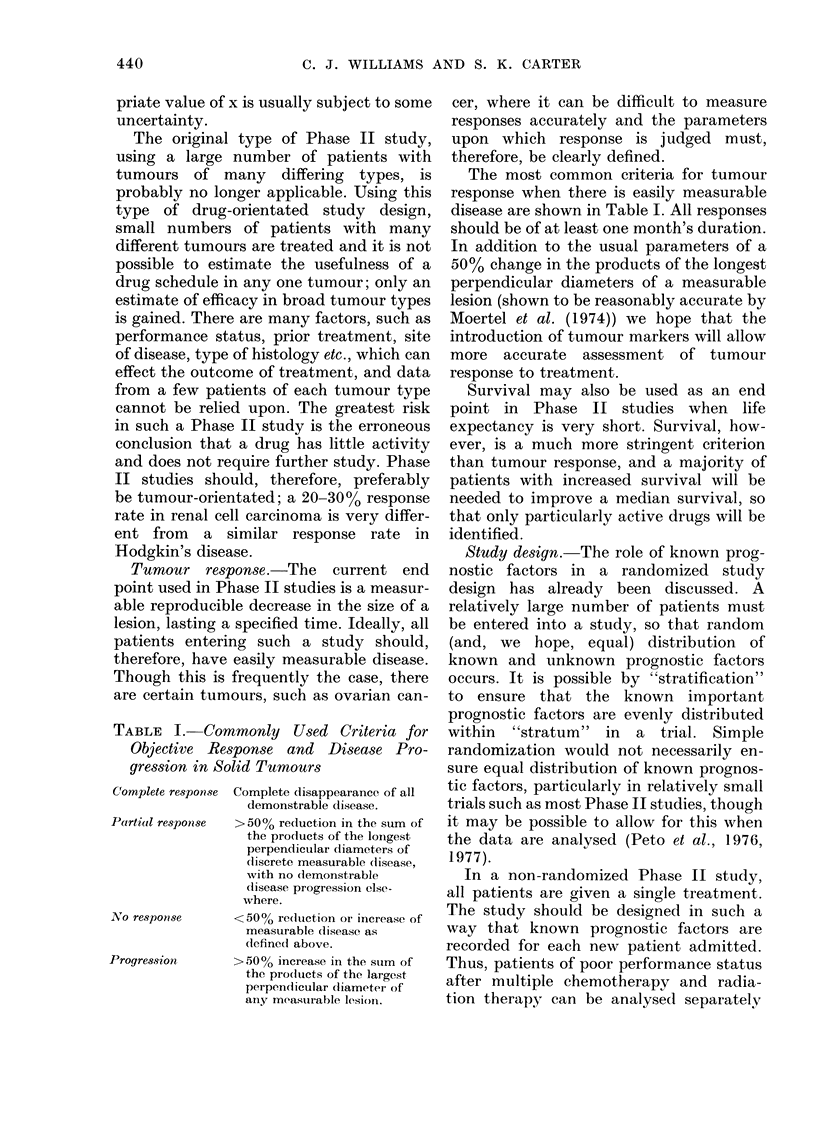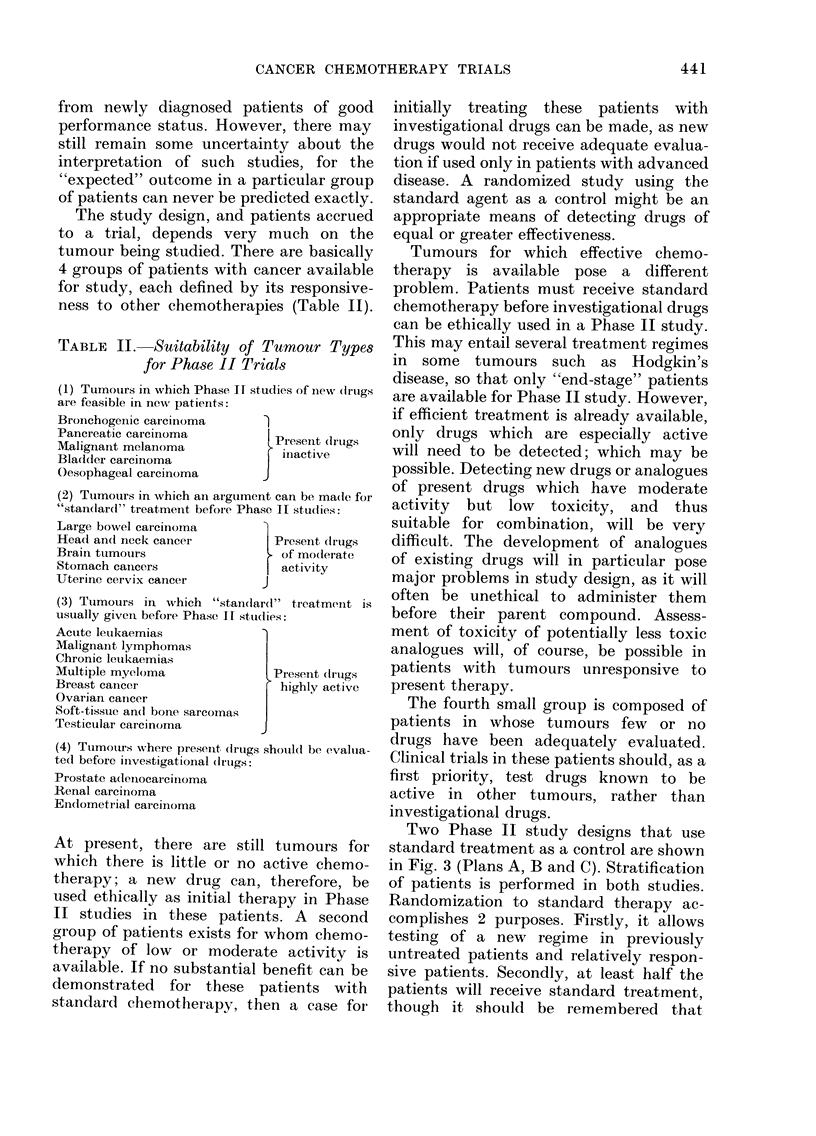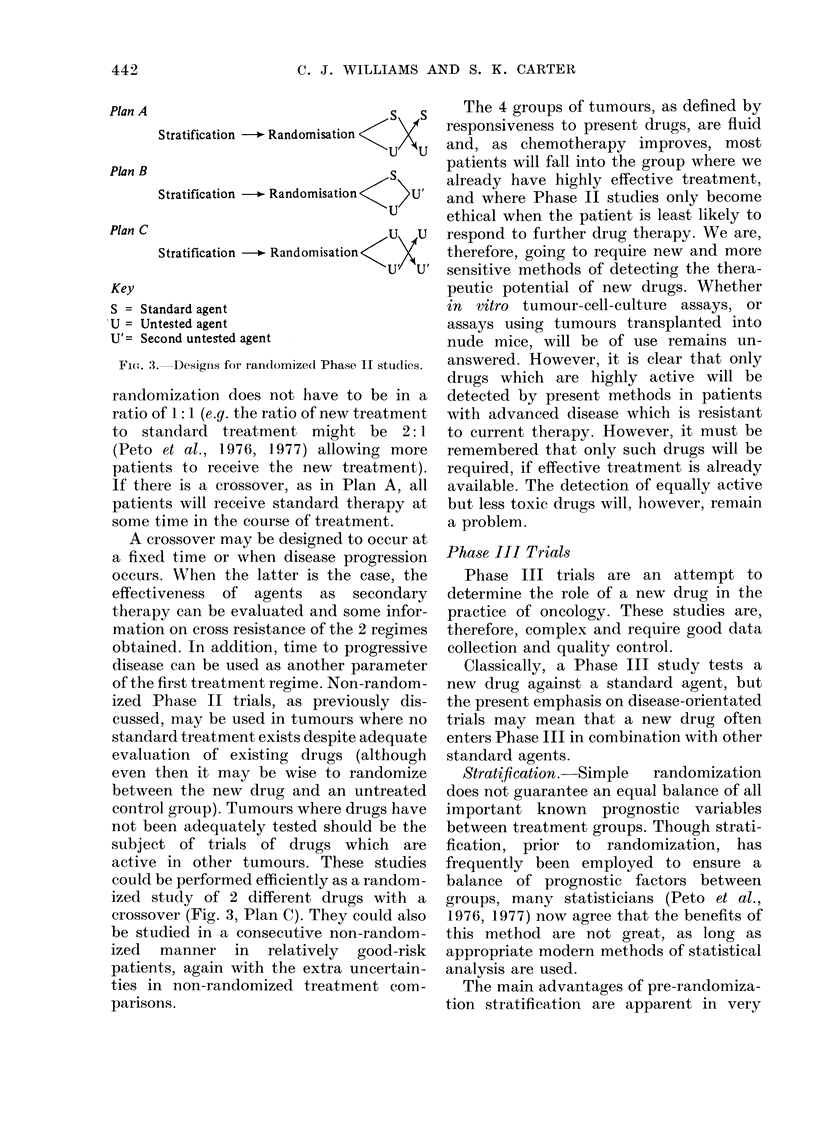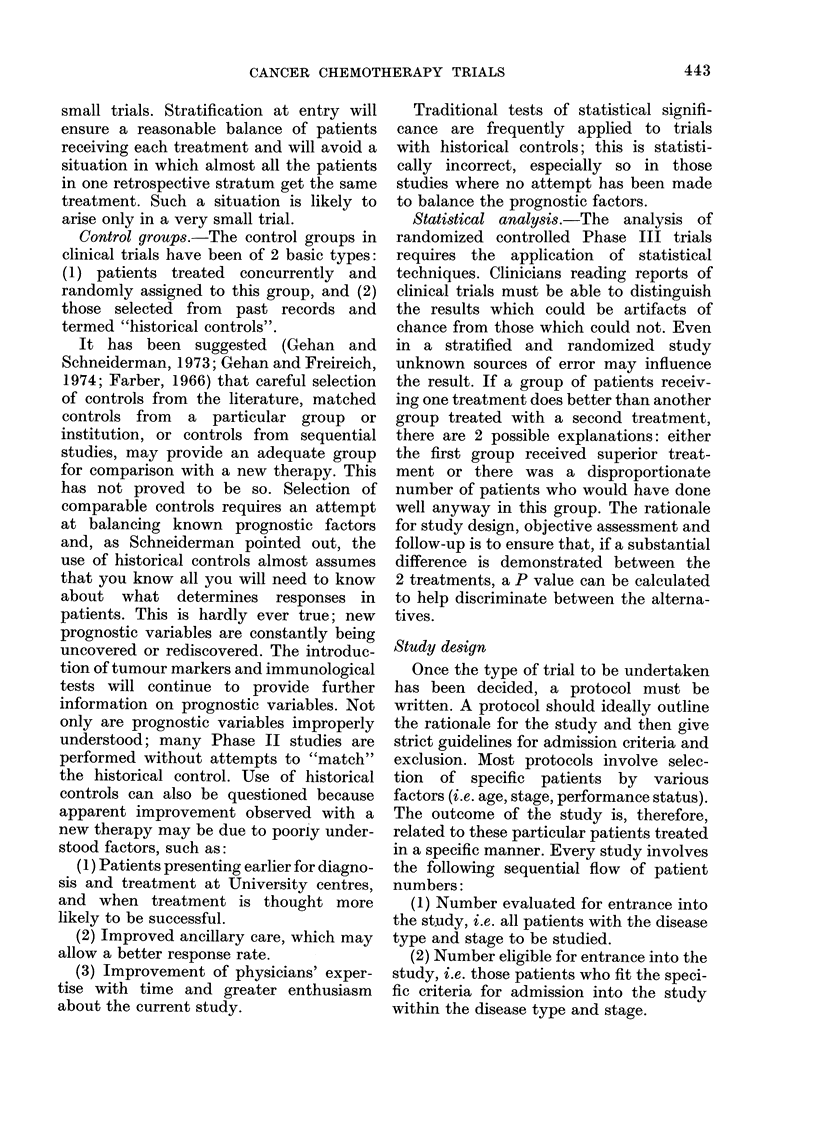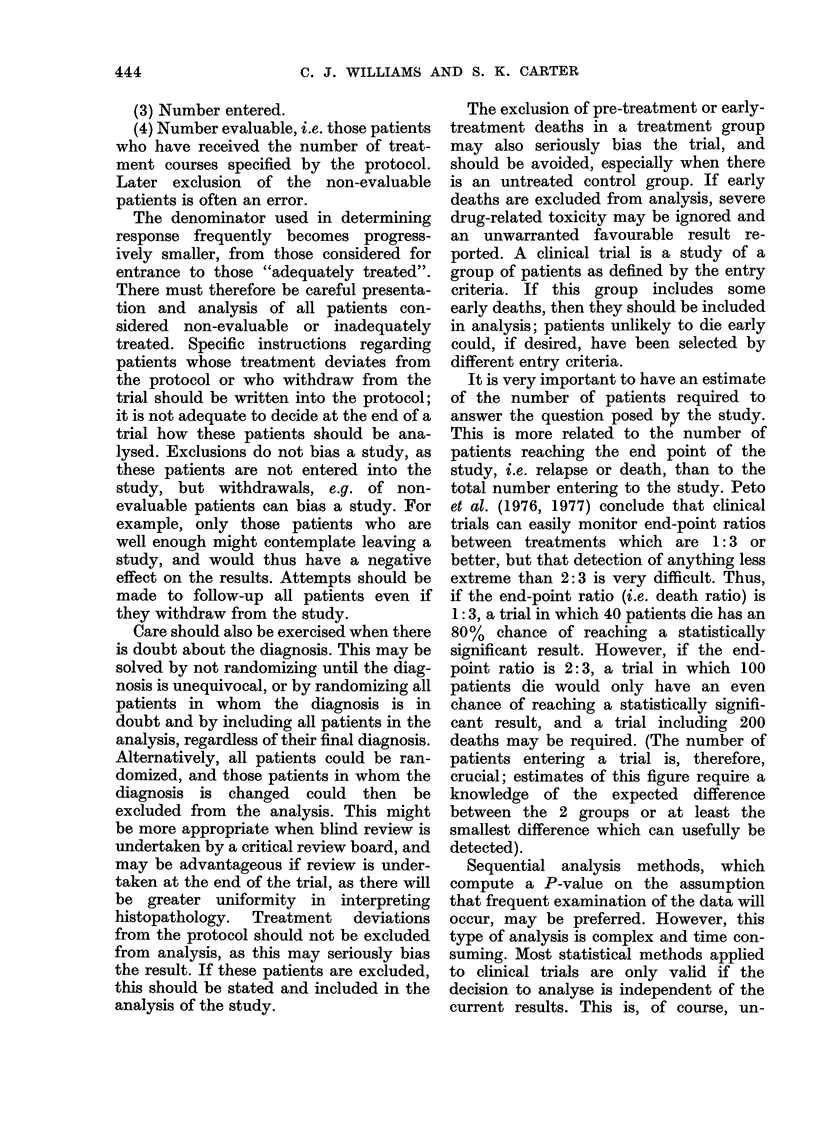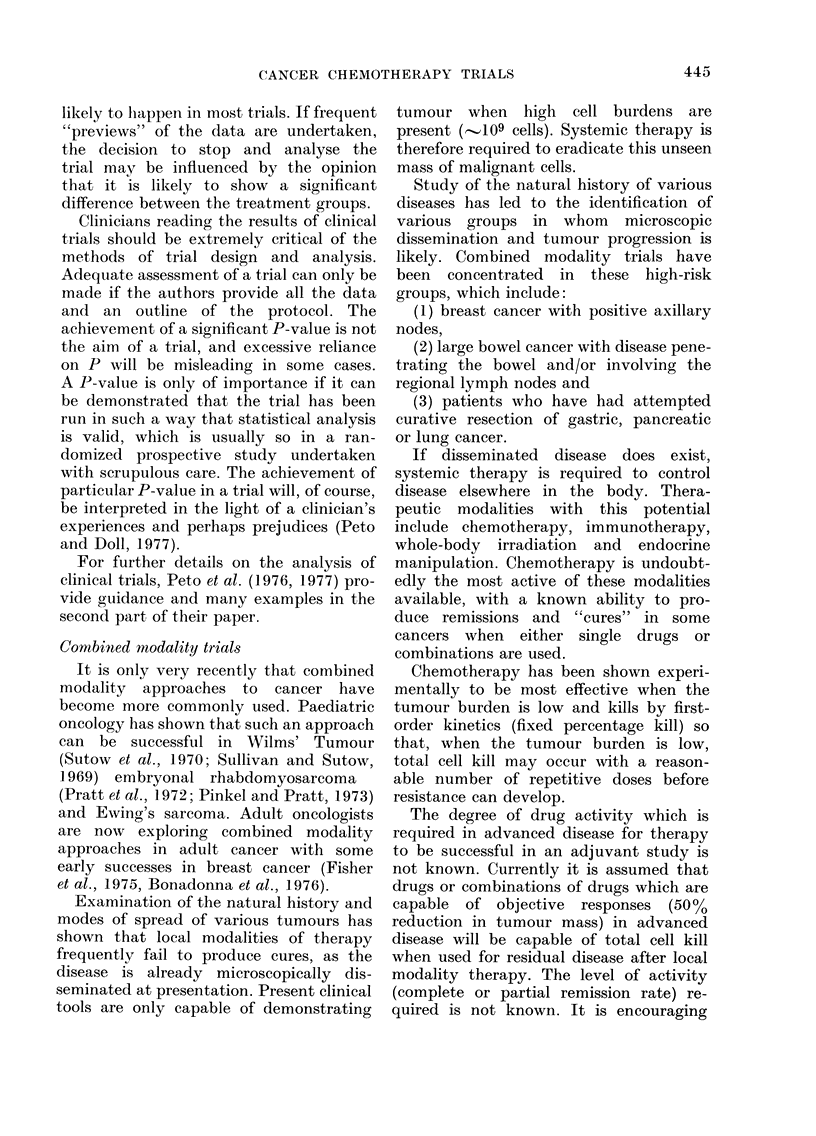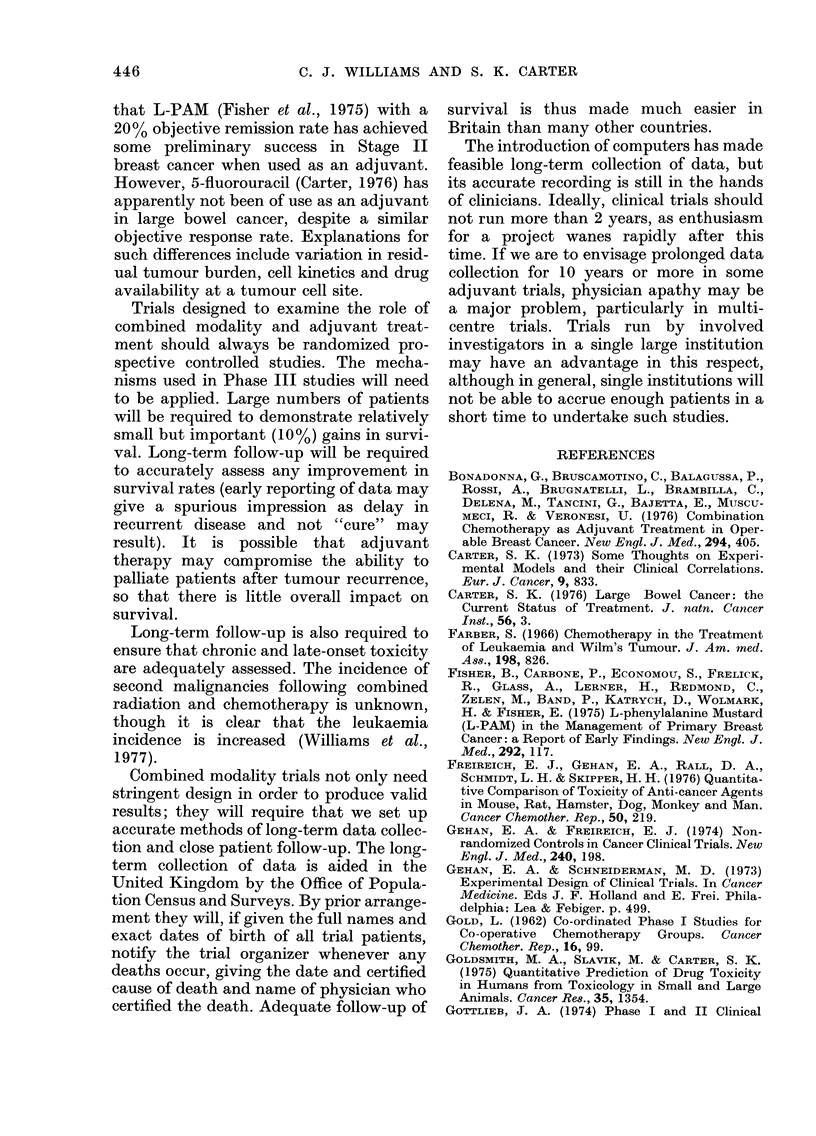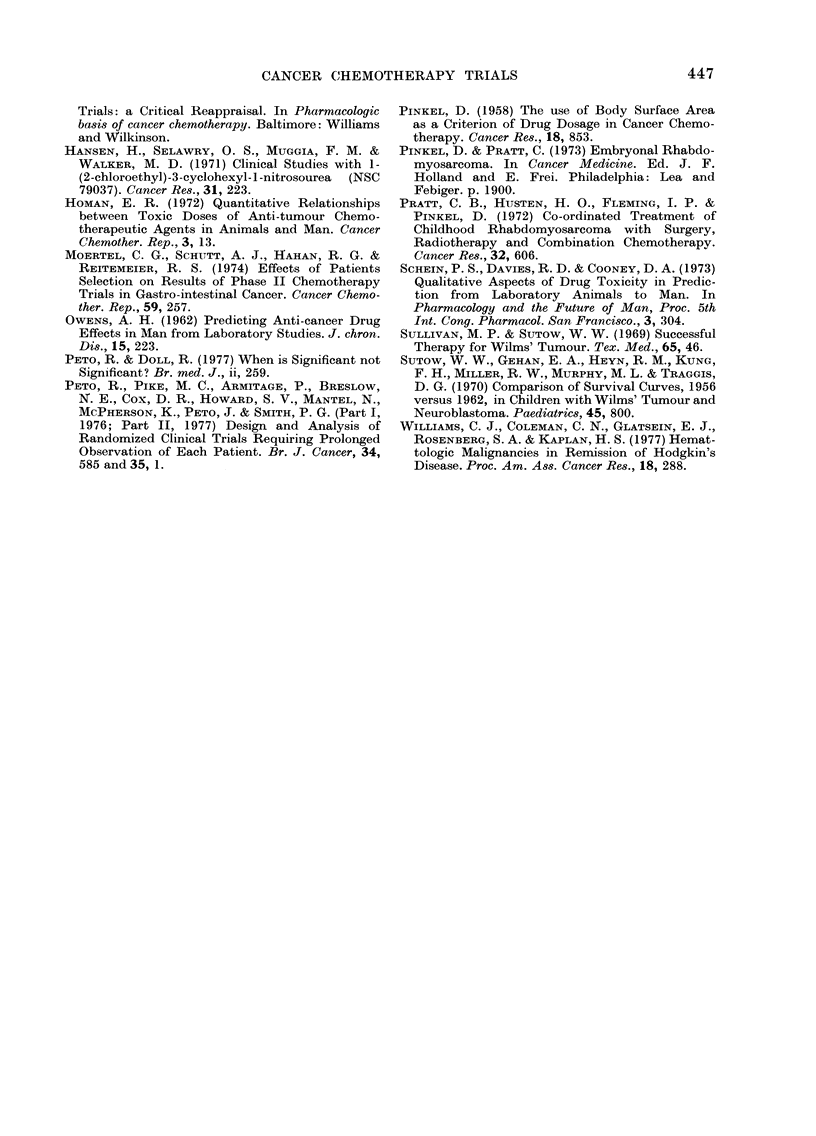# Management of trials in the development of cancer chemotherapy.

**DOI:** 10.1038/bjc.1978.64

**Published:** 1978-03

**Authors:** C. J. Williams, S. K. Carter

## Abstract

Potential anti-cancer agents have classically undergone clinical assessment in Phase I, II and III trials. This paper examines the role of these trials and pre-clinical studies in the light of improving cancer chemotherapy. Many patients must now be treated with standard therapy before investigational drugs can be ethically used. The introduction of combined modality trials will require a very prolonged follow-up to demonstrate improved survival and recognize late onset of chronic toxicity.


*Br. J. Cancer* (1978) **37**, 434

### MANAGEMENT OF TRIALS IN THE DEVELOPMENT OF CANCER CHEMOTHERAPY

C. J. WILLIAMS* AND S. K. CARTER†

*From the Stanford University Medical Center, Palo Alto, California*

Received 5 September 1977  Accepted 25 November 1977

**Summary.—Potential anti-cancer agents have classically undergone clinical assessment in Phase I, II and III trials. This paper examines the role of these trials and preclinical studies in the light of improving cancer chemotherapy. Many patients must now be treated with standard therapy before investigational drugs can be ethically used. The introduction of combined modality trials will require a very prolonged follow-up to demonstrate improved survival and recognize late onset of chronic toxicity.**

CANCER chemotherapy has made many advances in the treatment of malignant diseases. It is paradoxical that the often dramatic improvements in the treatment of these patients have now led to our present difficulties in the development of new agents.

In the 25 years since the National Cancer Institute modified the FDA-derived Phase I, II and III trials it has often become difficult to test new drugs, as they may only ethically be given to patients who have failed standard therapy.

At the time that these methods came into use, drug trials were simple clinical experiments in which some evidence of activity as a single agent was the end point. Most drugs tested showed low or moderate levels of activity, producing only minimal clinical responses. However, drugs are now combined so that they are more effective and are capable of producing frequent good responses and complete remissions in a number of tumours.

These improvements in chemotherapy have frequently made it difficult to test new drugs adequately. Many of the patients who can ethically be used in Phase I and II clinical trials are of low performance status, having received previous chemotherapy and/or radiotherapy, and are the patients who could be least expected to respond to further treatment. Thus, as we improve chemotherapy, it will become more difficult to test new drugs or analogues of existing ones, as many patients must be given standard treatment before we can ethically embark on treatments of unknown efficacy.

The situation is further complicated by the introduction of combined-modality therapy which includes chemotherapy. Though the methodology of such trials is still being worked out, it is already clear that these trials will be a major undertaking, requiring prolonged follow-up of patients for 10–20 years, if we are to learn the true impact of treatment on survival and the incidence of chronic or late-onset complications.

### *Preclinical studies*

Preclinical studies are performed in various animals and *in vitro* systems to assess the potential activity and toxicity of new drugs. While these studies are important in the selection of potentially active agents it is the results of Phase I

* Fellow in Oncology, Stanford University Hospital, Supported by Cancer Research Campaign, London.
† Director, Northern California Cancer Program.
Reprint requests to Dr C. J. Williams, CRC Medical Oncology Unit, Centre Block CF93, Southampton General Hospital, Southampton SO9 4XY.

and II trials that determine whether a new drug will be fully assessed for clinical use.

*Experimental models*

The use of experimental models in the selection of "active" compounds introduces many problems, the major of those being the selection of model test systems which are able to identify compounds with a high likelihood of clinical activity, (Carter, 1973). Although all systems are capable of producing true or false positives and negatives, it is not possible to know the false negative rate in present experimental models, as drugs which are negative never come to clinical trial.

All screening programmes must involve compromise, and it is clear that some compounds which would be clinically active are not detected by the present model systems and never reach clinical trial.

Owing to the logistic problem of testing large numbers of compounds of unknown efficacy in potential new model systems, it has been the policy of most centres working in this field only to use drugs of known efficacy in the detection of new model systems. Unfortunately, these particular drugs will have been identified by present systems which will have missed potentially active drugs. Thus, new systems will tend to mimic systems already in use, and may also fail to identify those "active" drugs missed by present systems.

Fortunately, compounds of known activity were not predicted by one system, and this provides a heterogeneity that can be used in evaluating new model systems. For example, Fig. 1 illustrates an approach which tests a new system against a number of drugs found to be active in L1210 and an equal number of drugs inactive in L1210, but active in other predictive models. The new system would be highly desirable if it showed activity by all the L1210-inactive drugs (even if it indicated lack of activity for the L1210-active drugs). The opposite result would be unattractive as it would only mimic the L1210 system. Most systems fall between these two

| *L1210 Active* | *L1210 Inactive* |
|---|---|
| Cyclophosphamide | Vincristine |
| Adriamycin | Bleomycin |
| Methotrexate | Hexamethylmelamine |
| 5-Fluorouracil | Dactinomycin |
| Nitrosoureas | Vinblastine |
| Phenylalanine mustard | Streptozotocin |
| Cytosine arabinoside | Prednisone |
| 6-Mercaptopurine | Dibromomannitol |
| Imidazole carboxamide | Mithramycin |
| Hydrozyurea | Myleran |

Fig. 1.—Drug activity in L1210 as an approach to evaluating a new screening system.

extremes, but such an evaluation is helpful in testing a new experimental system. Another approach is to repeat a similar trial (Fig. 2) but to divide the drugs between those most active in haematologic malignancies and those most active in solid tumours. (It is of note that only 2 drugs, methotrexate and cyclophosphamide, appear in both lists.)

| *Top 10 leukaemia drugs* | *Top 10 solid-tumour drugs* |
|---|---|
| Vincristine | Cyclophosphamide |
| Prednisone | Adriamycin |
| Methotrexate | Methotrexate |
| 6-Mercaptopurine | 5-Fluorouracil |
| Cytosine arabinoside | Nitrosoureas |
| Daunorubicin | Fhenylalanine mustard |
| L-Asparaginase | Hexamethylmelamine |
| 6-Thioguanine | Mitomycin C |
| Cyclophosphamide | Bleomycin |
| Hydrozyurea | Dactinomycin |

Fig. 2.—Drug activity in leukaemia and solid tumours as an approach to evaluating a new screening system.

Combinations of these approaches may be useful in uncovering new experimental systems applicable to certain tumour types. These techniques have been used by the NCI in the development of the B16 melanoma, P-388 leukaemia and Lewis Lung carcinoma systems. Use of these techniques will, we hope, allow the development of models capable of identifying some active drugs which have been missed in the past, as mass screening of new drugs in new model systems would be too costly and slow to justify it as an alternative method.

*Toxicity*

The extrapolation of data from animal to man poses many difficulties, though we have learned to predict toxicity in man with reasonable accuracy. Studies (Owens, 1962; Schein, Davies and Cooney, 1973) retrospectively comparing toxicities in animal species and man have shown that human toxicities in the haematopoietic, gastro-intestinal, renal and hepatic systems can generally be predicted from data collected in animals. However, in the two most commonly used large animal species (dog and monkey) there is a tendency to over-predict toxicity, especially in the renal and hepatic systems. In many animals, organ system toxicities are obtained at severely toxic and lethal doses. This may account for some of the over-prediction, since drugs are not given at such high levels in man.

Skin, cardiac and peripheral-nervous-system toxicity are not well predicted, and the detection of central-nervous-system toxicity is dependent on the care with which animals are assessed neurologically.

Specific parameters of toxicity, such as leucopenia in the haematopoietic system, are less accurately predicted than broad organ toxicities. Thus, anaemia may be the predominant haematopoietic toxicity in animals, whilst leucopenia may predominate in man.

A number of studies (Freireich *et al.*, 1976, Homan, 1972; Pinkel, 1958) have shown that a better correlation exists between toxic doses among animal species and man when the dose is expressed in terms of body surface area ($mg/m^2$) rather than body weight (mg/kg). Toxicity according to the blood or plasma level achieved has not been correlated, and might prove to be a more accurate predictor of toxicity and activity.

Various investigators have tried to define a safe starting dose in man by correlating the toxic doses in animal species and man. Freireich *et al.* (1976) suggested that one-third of the maximum tolerated dose defined by the weighted estimate of 5 animal species (in $mg/m^2$) would be a safe starting dose for most chemotherapy trials. However, Homan (1972) estimated that complete reliance on the use of one-third of the MTD ("dose which produced only minimal reversible toxicity") of the most sensitive large animal species (usually dog or monkey) gives a 6% probability of a toxic starting dose in man.

In the most recent analysis of this type (Goldsmith, Slavik and Carter, 1975) one third of the "toxic dose low" (TDL $mg/m^2$) for the dog and one third of the $LD_{10}$ ($mg/m^2$) in mice was compared with the "commonly used clinical dose" in man. The TDL is defined as the "lowest dose to produce pathologic alterations in haematologic, chemical, clinical or morphologic parameters; doubling this dose produces no lethality". The mouse $LD_{10}$ is the dose that kills 10% of the animals. Although the "commonly used clinical dose" in man may not represent the MTD, it is the dose that generally produces toxicity and may yield therapeutic effects. This analysis by Goldsmith *et al.* (1975) was performed to see how often the "commonly used human dose" would have been exceeded if the starting dose had been based on the animal data. Identical dose schedules were frequently not used in animals and man, and cumulative doses from animal schedules were correlated to those used in man by a method described by Freireich *et al.* (1976). However, this conversion relies on toxicity being related to the cumulative dose, regardless of schedule and this assumption has not been validated. Using this method, Goldsmith *et al.* (1975) showed that reliance on the 1/3 TDL, as used in most NCI trials, would have resulted in a dose exceeding the "commonly used human dose" in 5/30 (17%) of the trials. Thus, strict adherence to the 1/3 TDL guideline may not be appropriate for all drugs. Use of 1/3 $LD_{10}$ in mice as a starting dose would have exceeded the "commonly used human dose" in only 2 out of 19 trials (11%). Though these data are not sufficient to imply that the mouse offers a greater degree of predictability than large animal species, this evaluation is continuing.

*Antitumour activity*

Though screening for activity of new drugs in various transplantable and carcinogen-induced animal tumours does take place, there is no sure correlation between activity in these systems and in human tumours. It remains to be seen whether activity in human tumours can be more accurately predicted in human tumours transplanted to nude mice. If this were possible, it might improve the initial screening for new drugs and even allow screening for active drugs in individual tumours.

Many animal models have high labelling indices, though some recent systems, such as the Lewis lung and B16 melanoma, more closely mimic various human solid tumours with low labelling indices. Most importantly, we do not know whether these systems are failing to detect active drugs, only those drugs which show activity in animal models being entered into clinical trials.

The preclinical evaluation of new drugs should also include the development of methods of measuring tissue and blood levels of the drug; these methods should preferably be chemical rather than biological. As previously noted, the blood level achieved may be much more accurate in predicting toxicity and clinical activity than a dose based on surface area or body weight. The ability to measure drug levels will also assist in obtaining as much information as possible on absorption, mechanisms of metabolism and routes of excretion. Ideally, these pharmacological data should be available from animal studies prior to a drug being given in a Phase I study. In addition, useful information on drug interactions might be obtained, though this has rarely been done in the past.

*Clinical studies*

All clinical studies should consist of an ethically designed experiment which sets out to answer one or more specific questions. For a clinical study to be successful, a written protocol is required, as there are many different factors to be taken into account. These include, at least, decisions on the treatments to be studied, admission and exclusion criteria, randomization and stratification, deviations from protocol, exclusions and withdrawals, and the number of patients required to reach a valid conclusion. All clinical trials in drug development should ideally be prospective A successful clinical trial is one that reaches the correct conclusion, not one that produces a positive result.

*Phase I Trials*

A Phase I trial is essentially a clinical pharmacological evaluation to establish the maximum tolerated dose of a new drug by a given schedule and route of administration. It also identifies toxicity patterns and whether toxicity is predictable, tolerable and reversible. A Phase I trial is a study of therapeutic intent, but lack of evidence of antitumour activity should not be relied upon in the decision on whether to proceed to a Phase II study. Many of the patients who receive drugs in Phase I studies are from a selected population who are most unlikely to respond to therapy. Investigators should not be discouraged when a new drug shows little or no activity in this setting: the antitumour activity of new drugs can only be correctly ascertained in Phase II and III studies.

When new anticancer agents are first administered in patients, there is frequently a lack of relevant data to suggest safe methods of setting up the study. Ideally, before beginning a Phase I study the following information should be available:

(1) A method (preferably chemical) of measuring the compound in blood or other body fluids.

(2) A knowledge of blood levels achieved by single, multiple and infusion techniques of administration in several animal species.

(3) A knowledge of the blood level of the compound in relation to toxicity and therapeutic action.

(4) The pharmacology of the drug should be known in animal species, so that the methods of administration and study of human pharmacology are optimal.

All too frequently, drugs go into clinical trials without this information available. A question which has caused a great deal of debate among medical oncologists is whether a Phase I clinical trial of a new drug should be deferred until the pharmacological methodology has been developed. This could delay clinical studies for a long time. On the other hand, premature clinical assessment may run the risk of missing good drugs because they have been given in an inappropriate schedule, perhaps producing unnecessary toxicity.

*Dose escalation.*—One of the most critical steps in setting up a Phase I study is deciding on a starting dose and a method of dose escalation which allows the maximum tolerated dose (MTD) to be reached safely and efficiently. One of the more common methods used is the modified Fibonacci search scheme (Hansen *et al.*, 1971) which employs a fixed series of dose escalations with decreasing increments. In the absence of toxicity the dose escalations continue according to the scheme. However, when mild but reproducible toxicity is encountered, the escalation is reduced to the 30–35% level until the MTD is established. It has been estimated that this method would require 5 ($\pm 3$) dose escalations for most drugs. Goldsmith *et al.* (1975) estimated the number of escalations required to reach the "commonly used clinical dose" for 30 compounds starting from 1/3 TDL for dogs. Only 3 agents (10%) would have required more than 8 steps if the modified Fibonacci search were employed. There are several other methods in use. Gottlieb (1974) described a method starting at 1/3 TLD; he increased the dose by 50% increments and decreased to 25% increments when toxicity was encountered. Gold (1962) has proposed a geometric progression of dose escalations, starting at 1/100 the rodent $LD_{50}$, but with its progressively increasing doses per escalation this method would probably result in excessive toxicity for most compounds.

Currently, no statistical model for dose escalation is inherently safe and efficient for all drugs. Most investigators begin with large increments (rarely more than two-fold) when no toxicity is seen, and decrease the escalations when minimal toxicity is encountered. Currently, it is the opinion of many workers that it is safe to continue to double the dose as long as no toxicity has been observed at the previous level.

Dose escalation methods have not taken into account blood levels of the compound and correlated this with the toxicity associated with similar levels in animal models. Such techniques might allow safer and more efficient dose escalation, should a reproducible correlation exist between toxicity and blood levels in human and animal models.

In most trials, 3 patients are placed on the initial dose and adequate time is allowed for follow-up of delayed toxicity before entering patients on the next dose. As toxicity is encountered, more patients are entered at each dose level until the MTD is reached. Many investigators feel that doses should not be escalated in individual patients, as it becomes impossible to know whether a toxic effect is the result of the cumulative dose or the administered dose.

The goal of a Phase I study is to find the MTD for a particular drug schedule. In practice the MTD achieved in any trial is a function of the degree of prior therapy to which the patients have been exposed and their performance status. Patients of low performance status who have received extensive prior chemotherapy or radiation therapy, will have less tolerance for further chemotherapy. However, in the experience of the authors there have been no cases where the MTD derived in a Phase I study has been increased by a subsequent Phase II study. This may be related to the expertise and support facilities available to those who conduct Phase I studies, and to the natural caution of investigators in Phase II and III studies.

Phase I studies have essentially been trials of toxicity, the end point being the identification of the MTD. These studies are also an ideal opportunity to gather pharmacokinetic data. If these data are collected, then selection of dose schedules and methods of reducing toxicity could be the result of intelligent use of the data rather than the use of routine screening procedures.

*Phase II Trials*

Phase II trials are screening studies to determine whether a new drug has anti-tumour activity worthy of further clinical evaluation. A Phase II trial is not planned to define the ultimate role of the drug under investigation. Many Phase II trials use the maximum tolerated dose and schedule derived from Phase I studies; each Phase II study being a trial of the particular dose and schedule used.

Animal studies have shown that the therapeutic effects of many agents depend on the dose schedule used. Unfortunately, it has not been possible to use different doses and schedules in many Phase II studies. The danger in the use of set dose schedules is that a drug may be judged relatively inactive when used in a particular schedule and thus, not receive further study. Such a drug might, however, have been active when used in a different schedule. In most present NCI trials, drugs are given on an intermittent schedule daily for 5 days or as a single dose, and it is rare for different schedules to be tested by a randomized comparison. Decisions on drug dose and schedule are thus made on the basis of non-random comparison of their results achieved by two routine scheduling methods. Indeed, a particular schedule may be adopted because it is that used in the first trial to show antitumour activity of a drug.

Greater care in trial design, and more attention to the pharmacokinetic data gained in preclinical and Phase I studies, might allow more rational choice of dose and schedule regimes, rather than using blind screening systems. Phase II studies comparing various dose and schedule regimes would undoubtedly be more complex, requiring some of the mechanisms previously employed in Phase III studies. However, deciding on the dose and schedule is clearly a very important step in the development of a new drug, and deserves to be treated in a less cavalier fashion than at present.

On the completion of a Phase II study a reasonable estimate of the efficacy of the drug used in a particular dose schedule should be available. In addition, further more definitive information on drug toxicities should be available. The decision to continue drug development in a Phase III study will be made on this data, which can be treated as a risk-benefit ratio.

*Tumour-orientated trials.*—When efficiency is the criterion for the advisability for further trials, Gehan and Schneiderman (1973) have pointed out that the decision to be reached is whether or not an agent can be effective in x% of patients or more. The answer can usually be reached in a relatively small number of patients. The value of x% will vary according to the efficacy of drugs that are already available for the treatment of a particular tumour, and in non-randomized studies the appropriate value of x is usually subject to some uncertainty.

The original type of Phase II study, using a large number of patients with tumours of many differing types, is probably no longer applicable. Using this type of drug-orientated study design, small numbers of patients with many different tumours are treated and it is not possible to estimate the usefulness of a drug schedule in any one tumour; only an estimate of efficacy in broad tumour types is gained. There are many factors, such as performance status, prior treatment, site of disease, type of histology *etc.*, which can effect the outcome of treatment, and data from a few patients of each tumour type cannot be relied upon. The greatest risk in such a Phase II study is the erroneous conclusion that a drug has little activity and does not require further study. Phase II studies should, therefore, preferably be tumour-orientated; a 20–30% response rate in renal cell carcinoma is very different from a similar response rate in Hodgkin's disease.

*Tumour response.*—The current end point used in Phase II studies is a measurable reproducible decrease in the size of a lesion, lasting a specified time. Ideally, all patients entering such a study should, therefore, have easily measurable disease. Though this is frequently the case, there are certain tumours, such as ovarian cancer, where it can be difficult to measure responses accurately and the parameters upon which response is judged must, therefore, be clearly defined.

TABLE I.—*Commonly Used Criteria for Objective Response and Disease Progression in Solid Tumours*

| | |
|---|---|
| *Complete response* | Complete disappearance of all demonstrable disease. |
| *Partial response* | >50% reduction in the sum of the products of the longest perpendicular diameters of discrete measurable disease, with no demonstrable disease progression elsewhere. |
| *No response* | <50% reduction or increase of measurable disease as defined above. |
| *Progression* | >50% increase in the sum of the products of the largest perpendicular diameter of any measurable lesion. |

The most common criteria for tumour response when there is easily measurable disease are shown in Table I. All responses should be of at least one month's duration. In addition to the usual parameters of a 50% change in the products of the longest perpendicular diameters of a measurable lesion (shown to be reasonably accurate by Moertel *et al.* (1974)) we hope that the introduction of tumour markers will allow more accurate assessment of tumour response to treatment.

Survival may also be used as an end point in Phase II studies when life expectancy is very short. Survival, however, is a much more stringent criterion than tumour response, and a majority of patients with increased survival will be needed to improve a median survival, so that only particularly active drugs will be identified.

*Study design.*—The role of known prognostic factors in a randomized study design has already been discussed. A relatively large number of patients must be entered into a study, so that random (and, we hope, equal) distribution of known and unknown prognostic factors occurs. It is possible by "stratification" to ensure that the known important prognostic factors are evenly distributed within "stratum" in a trial. Simple randomization would not necessarily ensure equal distribution of known prognostic factors, particularly in relatively small trials such as most Phase II studies, though it may be possible to allow for this when the data are analysed (Peto *et al.*, 1976, 1977).

In a non-randomized Phase II study, all patients are given a single treatment. The study should be designed in such a way that known prognostic factors are recorded for each new patient admitted. Thus, patients of poor performance status after multiple chemotherapy and radiation therapy can be analysed separately from newly diagnosed patients of good performance status. However, there may still remain some uncertainty about the interpretation of such studies, for the "expected" outcome in a particular group of patients can never be predicted exactly.

The study design, and patients accrued to a trial, depends very much on the tumour being studied. There are basically 4 groups of patients with cancer available for study, each defined by its responsiveness to other chemotherapies (Table II).

TABLE II.—*Suitability of Tumour Types for Phase II Trials*

| | |
|---|---|
| (1) Tumours in which Phase II studies of new drugs are feasible in new patients: | |
| Bronchogenic carcinoma<br>Pancreatic carcinoma<br>Malignant melanoma<br>Bladder carcinoma<br>Oesophageal carcinoma | Present drugs inactive |
| (2) Tumours in which an argument can be made for "standard" treatment before Phase II studies: | |
| Large bowel carcinoma<br>Head and neck cancer<br>Brain tumours<br>Stomach cancers<br>Uterine cervix cancer | Present drugs of moderate activity |
| (3) Tumours in which "standard" treatment is usually given before Phase II studies: | |
| Acute leukaemias<br>Malignant lymphomas<br>Chronic leukaemias<br>Multiple myeloma<br>Breast cancer<br>Ovarian cancer<br>Soft-tissue and bone sarcomas<br>Testicular carcinoma | Present drugs highly active |
| (4) Tumours where present drugs should be evaluated before investigational drugs: | |
| Prostate adenocarcinoma<br>Renal carcinoma<br>Endometrial carcinoma | |

At present, there are still tumours for which there is little or no active chemotherapy; a new drug can, therefore, be used ethically as initial therapy in Phase II studies in these patients. A second group of patients exists for whom chemotherapy of low or moderate activity is available. If no substantial benefit can be demonstrated for these patients with standard chemotherapy, then a case for initially treating these patients with investigational drugs can be made, as new drugs would not receive adequate evaluation if used only in patients with advanced disease. A randomized study using the standard agent as a control might be an appropriate means of detecting drugs of equal or greater effectiveness.

Tumours for which effective chemotherapy is available pose a different problem. Patients must receive standard chemotherapy before investigational drugs can be ethically used in a Phase II study. This may entail several treatment regimes in some tumours such as Hodgkin's disease, so that only "end-stage" patients are available for Phase II study. However, if efficient treatment is already available, only drugs which are especially active will need to be detected; which may be possible. Detecting new drugs or analogues of present drugs which have moderate activity but low toxicity, and thus suitable for combination, will be very difficult. The development of analogues of existing drugs will in particular pose major problems in study design, as it will often be unethical to administer them before their parent compound. Assessment of toxicity of potentially less toxic analogues will, of course, be possible in patients with tumours unresponsive to present therapy.

The fourth small group is composed of patients in whose tumours few or no drugs have been adequately evaluated. Clinical trials in these patients should, as a first priority, test drugs known to be active in other tumours, rather than investigational drugs.

Two Phase II study designs that use standard treatment as a control are shown in Fig. 3 (Plans A, B and C). Stratification of patients is performed in both studies. Randomization to standard therapy accomplishes 2 purposes. Firstly, it allows testing of a new regime in previously untreated patients and relatively responsive patients. Secondly, at least half the patients will receive standard treatment, though it should be remembered that

*Key*
S = Standard agent
U = Untested agent
U' = Second untested agent

FIG. 3.—Designs for randomized Phase II studies.

randomization does not have to be in a ratio of 1 : 1 (*e.g.* the ratio of new treatment to standard treatment might be 2:1 (Peto *et al.*, 1976, 1977) allowing more patients to receive the new treatment). If there is a crossover, as in Plan A, all patients will receive standard therapy at some time in the course of treatment.

A crossover may be designed to occur at a fixed time or when disease progression occurs. When the latter is the case, the effectiveness of agents as secondary therapy can be evaluated and some information on cross resistance of the 2 regimes obtained. In addition, time to progressive disease can be used as another parameter of the first treatment regime. Non-randomized Phase II trials, as previously discussed, may be used in tumours where no standard treatment exists despite adequate evaluation of existing drugs (although even then it may be wise to randomize between the new drug and an untreated control group). Tumours where drugs have not been adequately tested should be the subject of trials of drugs which are active in other tumours. These studies could be performed efficiently as a randomized study of 2 different drugs with a crossover (Fig. 3, Plan C). They could also be studied in a consecutive non-randomized manner in relatively good-risk patients, again with the extra uncertainties in non-randomized treatment comparisons.

The 4 groups of tumours, as defined by responsiveness to present drugs, are fluid and, as chemotherapy improves, most patients will fall into the group where we already have highly effective treatment, and where Phase II studies only become ethical when the patient is least likely to respond to further drug therapy. We are, therefore, going to require new and more sensitive methods of detecting the therapeutic potential of new drugs. Whether *in vitro* tumour-cell-culture assays, or assays using tumours transplanted into nude mice, will be of use remains unanswered. However, it is clear that only drugs which are highly active will be detected by present methods in patients with advanced disease which is resistant to current therapy. However, it must be remembered that only such drugs will be required, if effective treatment is already available. The detection of equally active but less toxic drugs will, however, remain a problem.

### *Phase III Trials*

Phase III trials are an attempt to determine the role of a new drug in the practice of oncology. These studies are, therefore, complex and require good data collection and quality control.

Classically, a Phase III study tests a new drug against a standard agent, but the present emphasis on disease-orientated trials may mean that a new drug often enters Phase III in combination with other standard agents.

*Stratification.*—Simple randomization does not guarantee an equal balance of all important known prognostic variables between treatment groups. Though stratification, prior to randomization, has frequently been employed to ensure a balance of prognostic factors between groups, many statisticians (Peto *et al.*, 1976, 1977) now agree that the benefits of this method are not great, as long as appropriate modern methods of statistical analysis are used.

The main advantages of pre-randomization stratification are apparent in very small trials. Stratification at entry will ensure a reasonable balance of patients receiving each treatment and will avoid a situation in which almost all the patients in one retrospective stratum get the same treatment. Such a situation is likely to arise only in a very small trial.

*Control groups.*—The control groups in clinical trials have been of 2 basic types: (1) patients treated concurrently and randomly assigned to this group, and (2) those selected from past records and termed "historical controls".

It has been suggested (Gehan and Schneiderman, 1973; Gehan and Freireich, 1974; Farber, 1966) that careful selection of controls from the literature, matched controls from a particular group or institution, or controls from sequential studies, may provide an adequate group for comparison with a new therapy. This has not proved to be so. Selection of comparable controls requires an attempt at balancing known prognostic factors and, as Schneiderman pointed out, the use of historical controls almost assumes that you know all you will need to know about what determines responses in patients. This is hardly ever true; new prognostic variables are constantly being uncovered or rediscovered. The introduction of tumour markers and immunological tests will continue to provide further information on prognostic variables. Not only are prognostic variables improperly understood; many Phase II studies are performed without attempts to "match" the historical control. Use of historical controls can also be questioned because apparent improvement observed with a new therapy may be due to poorly understood factors, such as:

(1) Patients presenting earlier for diagnosis and treatment at University centres, and when treatment is thought more likely to be successful.

(2) Improved ancillary care, which may allow a better response rate.

(3) Improvement of physicians' expertise with time and greater enthusiasm about the current study.

Traditional tests of statistical significance are frequently applied to trials with historical controls; this is statistically incorrect, especially so in those studies where no attempt has been made to balance the prognostic factors.

*Statistical analysis.*—The analysis of randomized controlled Phase III trials requires the application of statistical techniques. Clinicians reading reports of clinical trials must be able to distinguish the results which could be artifacts of chance from those which could not. Even in a stratified and randomized study unknown sources of error may influence the result. If a group of patients receiving one treatment does better than another group treated with a second treatment, there are 2 possible explanations: either the first group received superior treatment or there was a disproportionate number of patients who would have done well anyway in this group. The rationale for study design, objective assessment and follow-up is to ensure that, if a substantial difference is demonstrated between the 2 treatments, a $P$ value can be calculated to help discriminate between the alternatives.

### *Study design*

Once the type of trial to be undertaken has been decided, a protocol must be written. A protocol should ideally outline the rationale for the study and then give strict guidelines for admission criteria and exclusion. Most protocols involve selection of specific patients by various factors (*i.e.* age, stage, performance status). The outcome of the study is, therefore, related to these particular patients treated in a specific manner. Every study involves the following sequential flow of patient numbers:

(1) Number evaluated for entrance into the study, *i.e.* all patients with the disease type and stage to be studied.

(2) Number eligible for entrance into the study, *i.e.* those patients who fit the specific criteria for admission into the study within the disease type and stage.

(3) Number entered.

(4) Number evaluable, *i.e.* those patients who have received the number of treatment courses specified by the protocol. Later exclusion of the non-evaluable patients is often an error.

The denominator used in determining response frequently becomes progressively smaller, from those considered for entrance to those "adequately treated". There must therefore be careful presentation and analysis of all patients considered non-evaluable or inadequately treated. Specific instructions regarding patients whose treatment deviates from the protocol or who withdraw from the trial should be written into the protocol; it is not adequate to decide at the end of a trial how these patients should be analysed. Exclusions do not bias a study, as these patients are not entered into the study, but withdrawals, *e.g.* of non-evaluable patients can bias a study. For example, only those patients who are well enough might contemplate leaving a study, and would thus have a negative effect on the results. Attempts should be made to follow-up all patients even if they withdraw from the study.

Care should also be exercised when there is doubt about the diagnosis. This may be solved by not randomizing until the diagnosis is unequivocal, or by randomizing all patients in whom the diagnosis is in doubt and by including all patients in the analysis, regardless of their final diagnosis. Alternatively, all patients could be randomized, and those patients in whom the diagnosis is changed could then be excluded from the analysis. This might be more appropriate when blind review is undertaken by a critical review board, and may be advantageous if review is undertaken at the end of the trial, as there will be greater uniformity in interpreting histopathology. Treatment deviations from the protocol should not be excluded from analysis, as this may seriously bias the result. If these patients are excluded, this should be stated and included in the analysis of the study.

The exclusion of pre-treatment or early-treatment deaths in a treatment group may also seriously bias the trial, and should be avoided, especially when there is an untreated control group. If early deaths are excluded from analysis, severe drug-related toxicity may be ignored and an unwarranted favourable result reported. A clinical trial is a study of a group of patients as defined by the entry criteria. If this group includes some early deaths, then they should be included in analysis; patients unlikely to die early could, if desired, have been selected by different entry criteria.

It is very important to have an estimate of the number of patients required to answer the question posed by the study. This is more related to the number of patients reaching the end point of the study, *i.e.* relapse or death, than to the total number entering to the study. Peto *et al.* (1976, 1977) conclude that clinical trials can easily monitor end-point ratios between treatments which are 1:3 or better, but that detection of anything less extreme than 2:3 is very difficult. Thus, if the end-point ratio (*i.e.* death ratio) is 1:3, a trial in which 40 patients die has an 80% chance of reaching a statistically significant result. However, if the end-point ratio is 2:3, a trial in which 100 patients die would only have an even chance of reaching a statistically significant result, and a trial including 200 deaths may be required. (The number of patients entering a trial is, therefore, crucial; estimates of this figure require a knowledge of the expected difference between the 2 groups or at least the smallest difference which can usefully be detected).

Sequential analysis methods, which compute a $P$-value on the assumption that frequent examination of the data will occur, may be preferred. However, this type of analysis is complex and time consuming. Most statistical methods applied to clinical trials are only valid if the decision to analyse is independent of the current results. This is, of course, unlikely to happen in most trials. If frequent "previews" of the data are undertaken, the decision to stop and analyse the trial may be influenced by the opinion that it is likely to show a significant difference between the treatment groups.

Clinicians reading the results of clinical trials should be extremely critical of the methods of trial design and analysis. Adequate assessment of a trial can only be made if the authors provide all the data and an outline of the protocol. The achievement of a significant $P$-value is not the aim of a trial, and excessive reliance on $P$ will be misleading in some cases. A $P$-value is only of importance if it can be demonstrated that the trial has been run in such a way that statistical analysis is valid, which is usually so in a randomized prospective study undertaken with scrupulous care. The achievement of particular $P$-value in a trial will, of course, be interpreted in the light of a clinician's experiences and perhaps prejudices (Peto and Doll, 1977).

For further details on the analysis of clinical trials, Peto *et al.* (1976, 1977) provide guidance and many examples in the second part of their paper.

*Combined modality trials*

It is only very recently that combined modality approaches to cancer have become more commonly used. Paediatric oncology has shown that such an approach can be successful in Wilms' Tumour (Sutow *et al.*, 1970; Sullivan and Sutow, 1969) embryonal rhabdomyosarcoma (Pratt *et al.*, 1972; Pinkel and Pratt, 1973) and Ewing's sarcoma. Adult oncologists are now exploring combined modality approaches in adult cancer with some early successes in breast cancer (Fisher *et al.*, 1975, Bonadonna *et al.*, 1976).

Examination of the natural history and modes of spread of various tumours has shown that local modalities of therapy frequently fail to produce cures, as the disease is already microscopically disseminated at presentation. Present clinical tools are only capable of demonstrating tumour when high cell burdens are present ($\sim 10^9$ cells). Systemic therapy is therefore required to eradicate this unseen mass of malignant cells.

Study of the natural history of various diseases has led to the identification of various groups in whom microscopic dissemination and tumour progression is likely. Combined modality trials have been concentrated in these high-risk groups, which include:

(1) breast cancer with positive axillary nodes,

(2) large bowel cancer with disease penetrating the bowel and/or involving the regional lymph nodes and

(3) patients who have had attempted curative resection of gastric, pancreatic or lung cancer.

If disseminated disease does exist, systemic therapy is required to control disease elsewhere in the body. Therapeutic modalities with this potential include chemotherapy, immunotherapy, whole-body irradiation and endocrine manipulation. Chemotherapy is undoubtedly the most active of these modalities available, with a known ability to produce remissions and "cures" in some cancers when either single drugs or combinations are used.

Chemotherapy has been shown experimentally to be most effective when the tumour burden is low and kills by first-order kinetics (fixed percentage kill) so that, when the tumour burden is low, total cell kill may occur with a reasonable number of repetitive doses before resistance can develop.

The degree of drug activity which is required in advanced disease for therapy to be successful in an adjuvant study is not known. Currently it is assumed that drugs or combinations of drugs which are capable of objective responses (50% reduction in tumour mass) in advanced disease will be capable of total cell kill when used for residual disease after local modality therapy. The level of activity (complete or partial remission rate) required is not known. It is encouraging that L-PAM (Fisher *et al.*, 1975) with a 20% objective remission rate has achieved some preliminary success in Stage II breast cancer when used as an adjuvant. However, 5-fluorouracil (Carter, 1976) has apparently not been of use as an adjuvant in large bowel cancer, despite a similar objective response rate. Explanations for such differences include variation in residual tumour burden, cell kinetics and drug availability at a tumour cell site.

Trials designed to examine the role of combined modality and adjuvant treatment should always be randomized prospective controlled studies. The mechanisms used in Phase III studies will need to be applied. Large numbers of patients will be required to demonstrate relatively small but important (10%) gains in survival. Long-term follow-up will be required to accurately assess any improvement in survival rates (early reporting of data may give a spurious impression as delay in recurrent disease and not "cure" may result). It is possible that adjuvant therapy may compromise the ability to palliate patients after tumour recurrence, so that there is little overall impact on survival.

Long-term follow-up is also required to ensure that chronic and late-onset toxicity are adequately assessed. The incidence of second malignancies following combined radiation and chemotherapy is unknown, though it is clear that the leukaemia incidence is increased (Williams *et al.*, 1977).

Combined modality trials not only need stringent design in order to produce valid results; they will require that we set up accurate methods of long-term data collection and close patient follow-up. The long-term collection of data is aided in the United Kingdom by the Office of Population Census and Surveys. By prior arrangement they will, if given the full names and exact dates of birth of all trial patients, notify the trial organizer whenever any deaths occur, giving the date and certified cause of death and name of physician who certified the death. Adequate follow-up of survival is thus made much easier in Britain than many other countries.

The introduction of computers has made feasible long-term collection of data, but its accurate recording is still in the hands of clinicians. Ideally, clinical trials should not run more than 2 years, as enthusiasm for a project wanes rapidly after this time. If we are to envisage prolonged data collection for 10 years or more in some adjuvant trials, physician apathy may be a major problem, particularly in multicentre trials. Trials run by involved investigators in a single large institution may have an advantage in this respect, although in general, single institutions will not be able to accrue enough patients in a short time to undertake such studies.

### REFERENCES

BONADONNA, G., BRUSCAMOTINO, C., BALAGUSSA, P., ROSSI, A., BRUGNATELLI, L., BRAMBILLA, C., DELENA, M., TANCINI, G., BAJETTA, E., MUSCUMECI, R. & VERONESI, U. (1976) Combination Chemotherapy as Adjuvant Treatment in Operable Breast Cancer. *New Engl. J. Med.*, **294**, 405.

CARTER, S. K. (1973) Some Thoughts on Experimental Models and their Clinical Correlations. *Eur. J. Cancer*, **9**, 833.

CARTER, S. K. (1976) Large Bowel Cancer: the Current Status of Treatment. *J. natn. Cancer Inst.*, **56**, 3.

FARBER, S. (1966) Chemotherapy in the Treatment of Leukaemia and Wilm's Tumour. *J. Am. med. Ass.*, **198**, 826.

FISHER, B., CARBONE, P., ECONOMOU, S., FRELICK, R., GLASS, A., LERNER, H., REDMOND, C., ZELEN, M., BAND, P., KATRYCH, D., WOLMARK, H. & FISHER, E. (1975) L-phenylalanine Mustard (L-PAM) in the Management of Primary Breast Cancer: a Report of Early Findings. *New Engl. J. Med.*, **292**, 117.

FREIREICH, E. J., GEHAN, E. A., RALL, D. A., SCHMIDT, L. H. & SKIPPER, H. H. (1976) Quantitative Comparison of Toxicity of Anti-cancer Agents in Mouse, Rat, Hamster, Dog, Monkey and Man. *Cancer Chemother. Rep.*, **50**, 219.

GEHAN, E. A. & FREIREICH, E. J. (1974) Non-randomized Controls in Cancer Clinical Trials. *New Engl. J. Med.*, **240**, 198.

GEHAN, E. A. & SCHNEIDERMAN, M. D. (1973) Experimental Design of Clinical Trials. In *Cancer Medicine*. Eds J. F. Holland and E. Frei. Philadelphia: Lea & Febiger. p. 499.

GOLD, L. (1962) Co-ordinated Phase I Studies for Co-operative Chemotherapy Groups. *Cancer Chemother. Rep.*, **16**, 99.

GOLDSMITH, M. A., SLAVIK, M. & CARTER, S. K. (1975) Quantitative Prediction of Drug Toxicity in Humans from Toxicology in Small and Large Animals. *Cancer Res.*, **35**, 1354.

GOTTLIEB, J. A. (1974) Phase I and II Clinical

Trials: a Critical Reappraisal. In *Pharmacologic basis of cancer chemotherapy*. Baltimore: Williams and Wilkinson.

HANSEN, H., SELAWRY, O. S., MUGGIA, F. M. & WALKER, M. D. (1971) Clinical Studies with 1-(2-chloroethyl)-3-cyclohexyl-1-nitrosourea (NSC 79037). *Cancer Res.*, **31**, 223.

HOMAN, E. R. (1972) Quantitative Relationships between Toxic Doses of Anti-tumour Chemotherapeutic Agents in Animals and Man. *Cancer Chemother. Rep.*, **3**, 13.

MOERTEL, C. G., SCHUTT, A. J., HAHAN, R. G. & REITEMEIER, R. S. (1974) Effects of Patients Selection on Results of Phase II Chemotherapy Trials in Gastro-intestinal Cancer. *Cancer Chemother. Rep.*, **59**, 257.

OWENS, A. H. (1962) Predicting Anti-cancer Drug Effects in Man from Laboratory Studies. *J. chron. Dis.*, **15**, 223.

PETO, R. & DOLL, R. (1977) When is Significant not Significant? *Br. med. J.*, ii, 259.

PETO, R., PIKE, M. C., ARMITAGE, P., BRESLOW, N. E., COX, D. R., HOWARD, S. V., MANTEL, N., MCPHERSON, K., PETO, J. & SMITH, P. G. (Part I, 1976; Part II, 1977) Design and Analysis of Randomized Clinical Trials Requiring Prolonged Observation of Each Patient. *Br. J. Cancer*, **34**, 585 and **35**, 1.

PINKEL, D. (1958) The use of Body Surface Area as a Criterion of Drug Dosage in Cancer Chemotherapy. *Cancer Res.*, **18**, 853.

PINKEL, D. & PRATT, C. (1973) Embryonal Rhabdomyosarcoma. In *Cancer Medicine*. Ed. J. F. Holland and E. Frei. Philadelphia: Lea and Febiger. p. 1900.

PRATT, C. B., HUSTEN, H. O., FLEMING, I. P. & PINKEL, D. (1972) Co-ordinated Treatment of Childhood Rhabdomyosarcoma with Surgery, Radiotherapy and Combination Chemotherapy. *Cancer Res.*, **32**, 606.

SCHEIN, P. S., DAVIES, R. D. & COONEY, D. A. (1973) Qualitative Aspects of Drug Toxicity in Prediction from Laboratory Animals to Man. In *Pharmacology and the Future of Man, Proc. 5th Int. Cong. Pharmacol. San Francisco.*, **3**, 304.

SULLIVAN, M. P. & SUTOW, W. W. (1969) Successful Therapy for Wilms' Tumour. *Tex. Med.*, **65**, 46.

SUTOW, W. W., GEHAN, E. A., HEYN, R. M., KUNG, F. H., MILLER, R. W., MURPHY, M. L. & TRAGGIS, D. G. (1970) Comparison of Survival Curves, 1956 versus 1962, in Children with Wilms' Tumour and Neuroblastoma. *Paediatrics*, **45**, 800.

WILLIAMS, C. J., COLEMAN, C. N., GLATSEIN, E. J., ROSENBERG, S. A. & KAPLAN, H. S. (1977) Hematologic Malignancies in Remission of Hodgkin's Disease. *Proc. Am. Ass. Cancer Res.*, **18**, 288.